# Pancreas-kidney transplantation: what every radiologist should know

**DOI:** 10.1590/0100-3984.2020.0108

**Published:** 2021

**Authors:** Alexandre Makoto Minoda, Fernando dos Santos Ferreira, Karllos Diego Ribeiro Santos, Cristiano de Souza Leão, Eduardo Just da Costa e Silva, Andréa Farias de Melo-Leite

**Affiliations:** 1 Instituto de Medicina Integral Professor Fernando Figueira (IMIP), Recife, PE, Brasil.; 2 Universidade Federal de Pernambuco (UFPE), Recife, PE, Brazil.

**Keywords:** Pancreas transplantation, Kidney transplantation, Organ transplantation, Diabetes mellitus, Postoperative complications, Transplante de pâncreas, Transplante de rim, Transplante de órgãos, Diabetes mellitus, Complicações pós-operatórias

## Abstract

Pancreas transplantation is a well-established treatment for patients with complicated diabetes mellitus and advanced renal failure. The most common procedure is simultaneous pancreas-kidney transplantation, in which the pancreas graft is positioned in the right pelvic region and the kidney graft is positioned in the left iliac fossa. Various imaging methods are used for the post-transplantation evaluation of the graft parenchyma and vascular anatomy, as well as for the identification of possible complications. As the number of cases increases, it is fundamental that radiologists understand the surgical procedure and the postoperative anatomy, as well as to recognize the possible postoperative complications and their imaging aspects, with the aim of providing the best guidance in the postoperative management of transplant recipients.

## Abstract (pt)

O transplante de pâncreas representa uma terapia bem estabelecida no tratamento de pacientes com diabetes mellitus complicada com insuficiência renal em estágios avançados. A maior parte ocorre em associação ao transplante renal (transplante simultâneo de pâncreas e rim), no qual se posiciona o enxerto pancreático na região pélvica direita e o enxerto renal na fossa ilíaca esquerda. Diversos métodos de imagem são utilizados para avaliação parenquimatosa dos enxertos, bem como a anatomia vascular e as possíveis complicações. A compreensão do procedimento cirúrgico, das técnicas utilizadas e da anatomia pós-cirúrgica é fundamental à medida que o número de casos aumenta, assim como conhecer as possíveis complicações associadas e seus aspectos de imagem, com a finalidade de proporcionar o melhor direcionamento no manejo pós-operatório de receptores de transplantes.

## INTRODUCTION

Pancreas transplantation is a well-established treatment for patients with complicated diabetes mellitus and advanced renal failure^(1)^. Simultaneous pancreas-kidney transplantation (SPKT), which is the most common procedure, has provided an increase in the life expectancy of such patients^(2-4)^. In addition to SPKT, isolated pancreas transplantation or pancreas transplantation after kidney transplantation, in separate procedures at different time points, can be performed^(2,4)^. Currently the most common form of pancreas transplantation, SPKT accounts for 75% of all pancreas transplantations in Brazil^(4,5)^.

Various imaging techniques can be used in order to detect early and late complications after pancreas transplantation^(1,2)^. The techniques used most often are ultrasound, computed tomography (CT), and magnetic resonance imaging (MRI).

## ANATOMY AND SURGICAL TECHNIQUE

**Figure 1:**
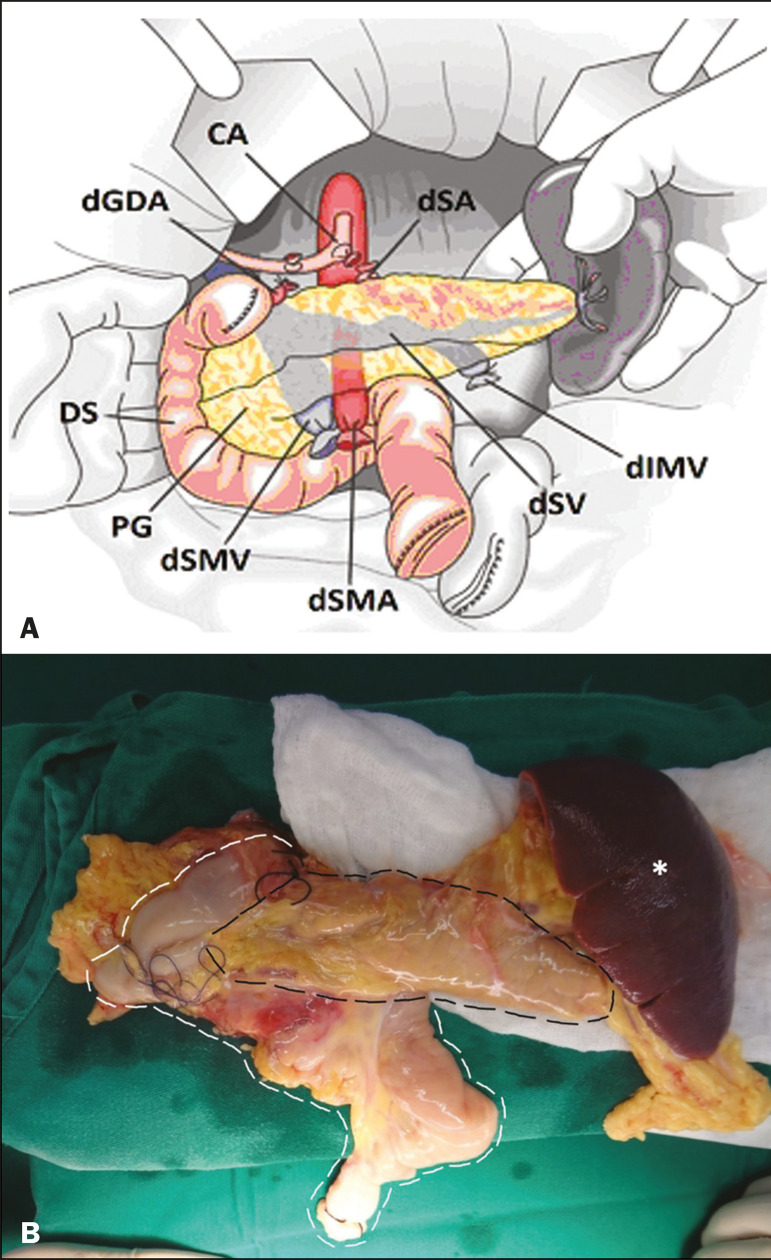
**A:** Schematic drawing (anterior view) showing the acquisition of a pancreatic graft (PG), removed together with its vascular support and one duodenal segment (DS) from the donor. For the purposes of transplantation, the donor gastroduodenal artery (dGDA), donor splenic artery (dSA), donor superior mesenteric artery (dSMA), donor superior mesenteric vein (dSMV) donor inferior mesenteric vein (dIMV), and donor splenic vein (dSV) must be connected and sectioned, as must the proximal segment of the portal vein. **B:** Photograph showing acquisition of the pancreatic duodenal block, the pancreatic allograft (black dotted line) being removed from the donor together with the duodenal segment (white dotted line), prior to excision of the spleen (asterisk). CA, celiac artery.

**Figure 2:**
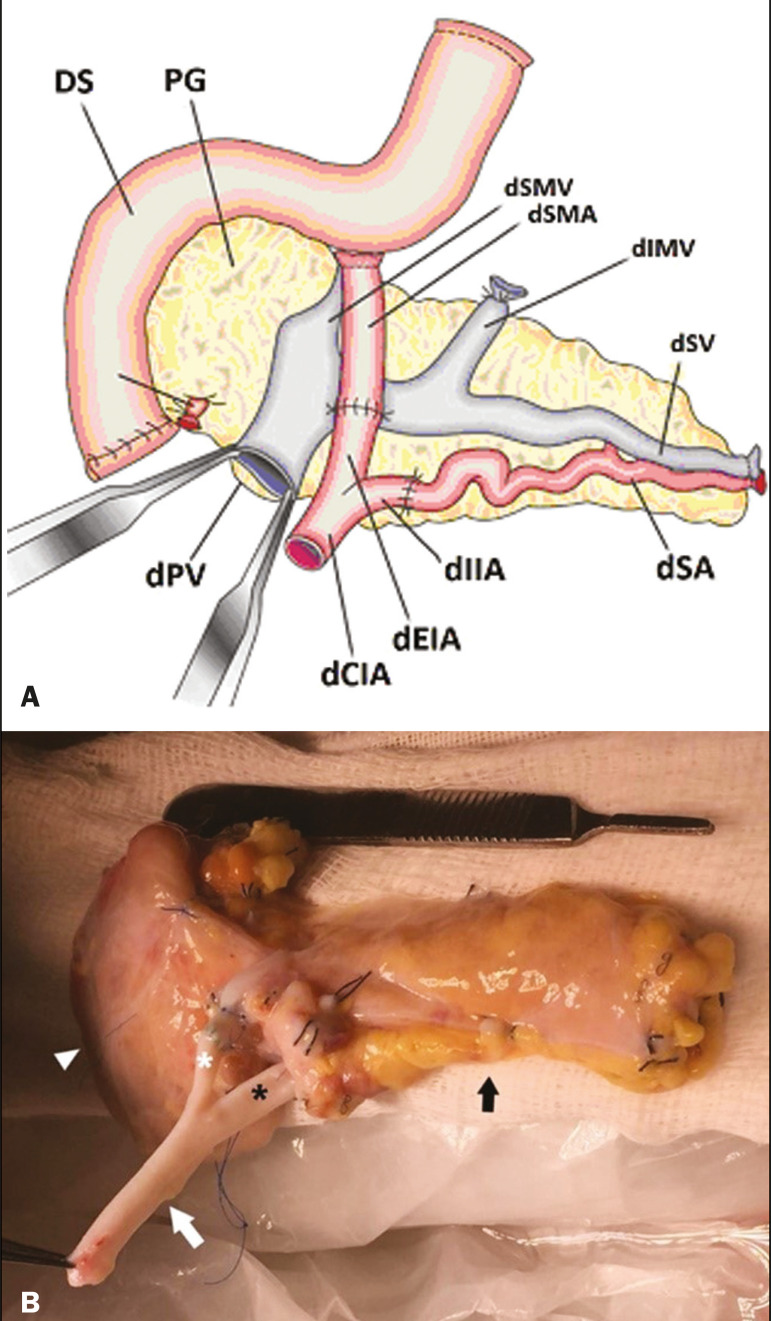
**A:** Schematic drawing showing a posterior view of the pancreatic graft (PG) and the duodenal segment (DS) of the donor, with an emphasis on the vascular anastomoses. End-to-end anastomosis between the donor external iliac artery (dEIA) and the donor superior mesenteric artery (dSMA) and between the donor internal iliac artery (dIIA) and the donor splenic artery (dSA) is performed, forming a Y graft. The donor common iliac artery (dCIA) serves as the common arterial channel of the pancreatic graft. The veins are resected together with a short segment of the portal vein (dPV), which is commonly used for venous anastomosis in the recipient. **B:** Pancreatic duodenal block with pancreatic graft (black arrow) and one duodenal segment (arrowhead) after anastomosis of the arterial Y graft, formed by the common iliac artery (white arrow), external iliac artery (white asterisk) and internal iliac artery (black asterisk) of the donor.

**Figure 3:**
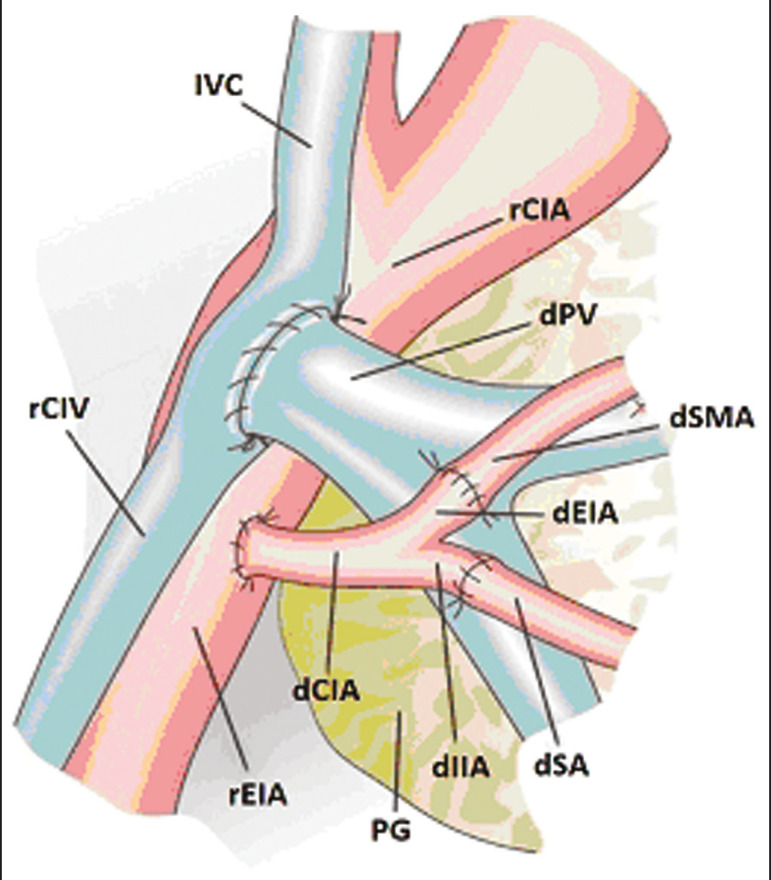
Schematic drawing showing vascular anastomoses after revascularization of the pancreatic graft (PG) in the recipient. End-to-side anastomosis is performed to join the arterial Y graft, via the donor common iliac artery (dCIA) to the recipient common iliac artery (rCIA) or the recipient external iliac artery (rEIA). End-to-side anastomosis is also used to connect the donor portal vein (dPV) to the right common iliac vein of the recipient (rCIV). IVC, inferior vena cava; dSMA, superior mesenteric artery; dIIA, internal iliac artery; dSA, splenic artery.

The most common procedure used in SPKT consists in positioning the pancreas graft in the right pelvic region and the kidney graft in the left iliac fossa during the same surgical procedure^(2,6,7)^. The pancreatic allograft is removed from the donor, together with the duodenum and vascular support (Figures 1A and 1B). The arterial blood supply to the pancreas comes from two main arteries: the superior mesenteric artery and the splenic artery^(2)^. The donor iliac arteries are also removed to form a Y graft, with an end-to-end anastomosis between the splenic artery and the superior mesenteric artery^(1,2)^, as depicted in Figures 2A and 2B. In the recipient, end-to-side anastomosis is performed between the arterial Y graft and the common iliac artery or the external iliac artery (Figure 3).

The venous drainage of the allograft consists of the intrapancreatic veins^(1,2)^, which drain into the splenic vein and the superior mesenteric vein, subsequently draining into the portal vein, which is used for venous anastomosis in the recipient (Figure 2A), allowing drainage into the systemic venous system (anastomosis in the common iliac vein or inferior vena cava) or the portal venous system (anastomosis in the superior mesenteric vein), as illustrated in Figure 3.

**Figure 4:**
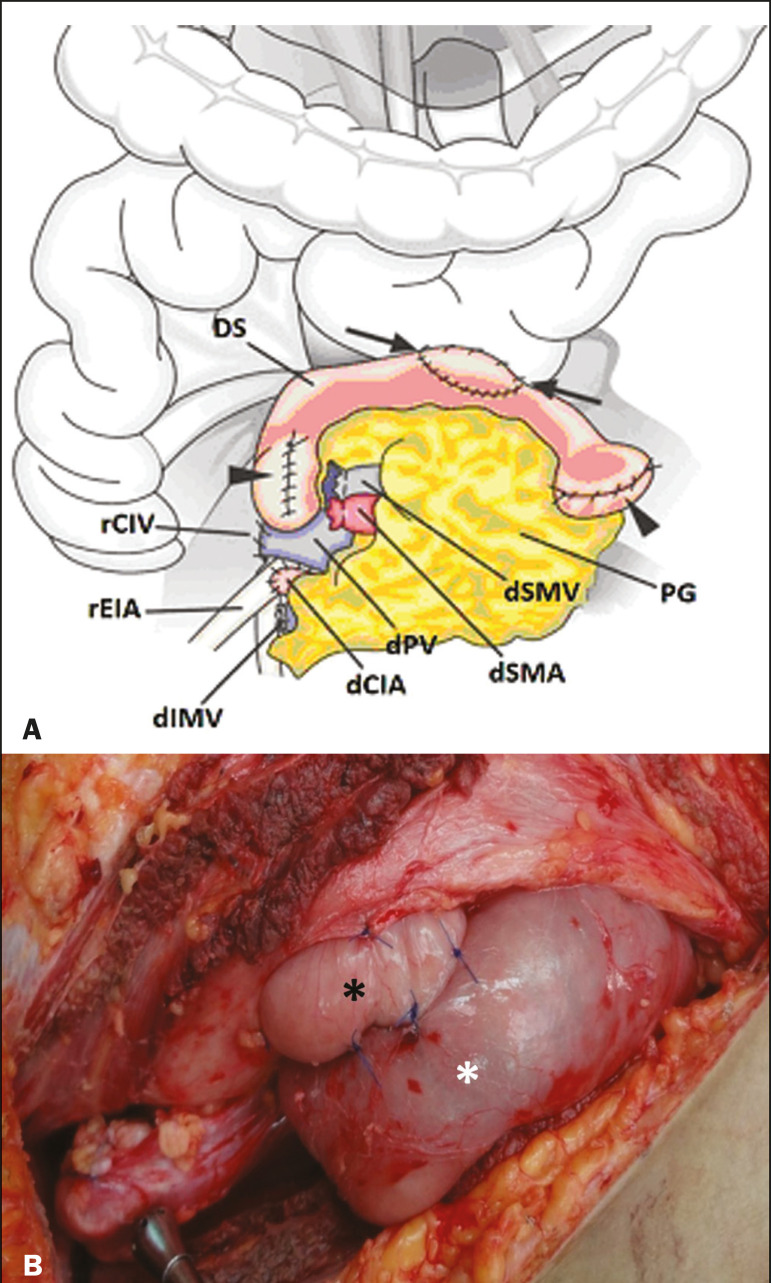
**A:** Schematic drawing illustrating the intraoperative appearance of the pancreatic graft (PG) positioned in the pelvis via side-to-side duodenojejunal anastomosis (arrows) linking the donor duodenal segment (DS) to the jejunum of the recipient. The donor duodenum is closed at both ends using sutures or staples (arrowheads). The native pancreas of the recipient remains in the upper abdomen. **B:** Intraoperative aspect of SPKT with enteric drainage of exocrine pancreatic secretions, by anastomosis between the donor duodenal stump (black asterisk) and the small intestine of the recipient (white asterisk), approximately 25 cm from the ileocecal valve. dIMV, inferior mesenteric vein; dSMV, superior mesenteric vein.

The exocrine pancreatic secretions can be drained to the gastrointestinal tract (Figures 4A and 4B) or to the bladder of the recipient^(1,2)^. Pancreas transplantation with enteric drainage is now more commonly performed and consists of anastomosis between the donor duodenal stump and the small intestine of the recipient^(1,2,6)^.

Regarding kidney transplantation, the donor renal artery is usually anastomosed end-to-side to the external or common iliac artery of the recipient and the renal vein is anastomosed to the external iliac vein of the recipient^(6,8)^.

## NORMAL IMAGING ASPECTS AFTER TRANSPLANTATION

On ultrasound, the normal appearance of the pancreas graft is that of a homogeneous hypoechoic structure, although Doppler ultrasound can demonstrate graft perfusion, as well as the vascular anatomy^(1,2)^. On spectral Doppler, flow velocity waveforms can be obtained from the superior mesenteric artery, splenic artery, and intrapancreatic duct, those waveforms being characterized by a rapid increase in systolic blood pressure and antegrade diastolic flow. Vascular resistance is low, usually with a resistance index between 0.5 and 0.7^(1,2)^. The venous structures demonstrate a monophasic waveform within an anechoic lumen^(2)^.

**Figure 5:**
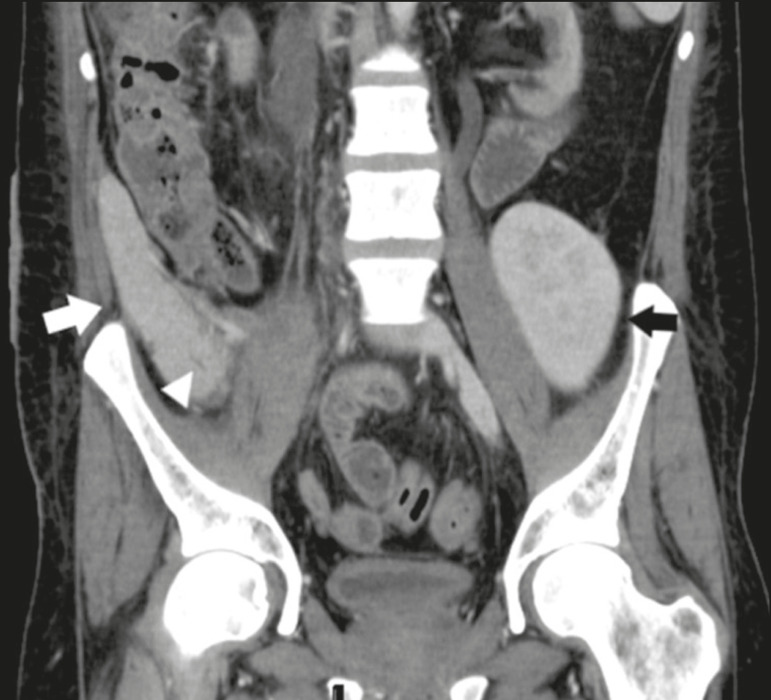
Coronal reconstruction of contrast-enhanced CT, showing normal parenchymal enhancement of the body and tail of the pancreatic graft (white arrow) in the right iliac fossa. A nondilated segment of the pancreatic duct is displayed (arrowhead). Note the renal graft with normal parenchymal enhancement (black arrow) in the left iliac fossa.

**Figure 6:**
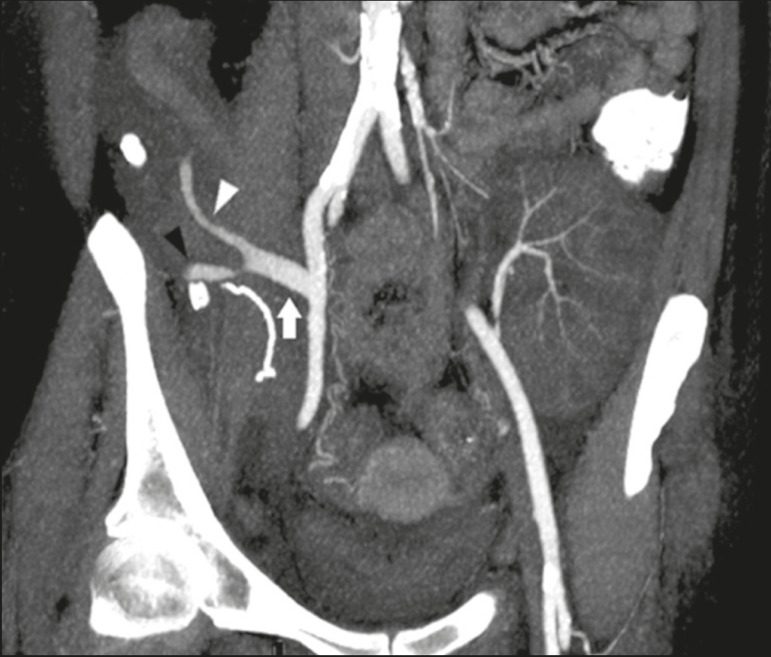
Maximum intensity projection image in the arterial phase of contrast-enhanced CT (coronal reconstruction), showing a patent Y graft (white arrow) anastomosed to the right external iliac artery. Note the anastomosis of the Y graft to the branches of the splenic artery (white arrowhead) and superior mesenteric artery (black arrowhead), with narrowing of the lumen of the superior mesenteric artery.

Unenhanced CT shows the pancreatic allograft as a homogeneous mass that is isoattenuating in relation to nondistended bowel, and the administration of contrast typically results in uniform parenchymal enhancement that is greater during the arterial phase (Figure 5), affording a better evaluation of the transplant anatomy^(1,2)^. We emphasize the arterial Y graft and the peripancreatic/intrapancreatic arterial vasculature, as well as the donor portal vein and its anastomosis^(1)^, as shown in Figure 6. On MRI, the normal pancreatic graft shows a signal that is isointense on T1-weighted images and hypointense on T2-weighted images^(1,2)^.

**Figure 7:**
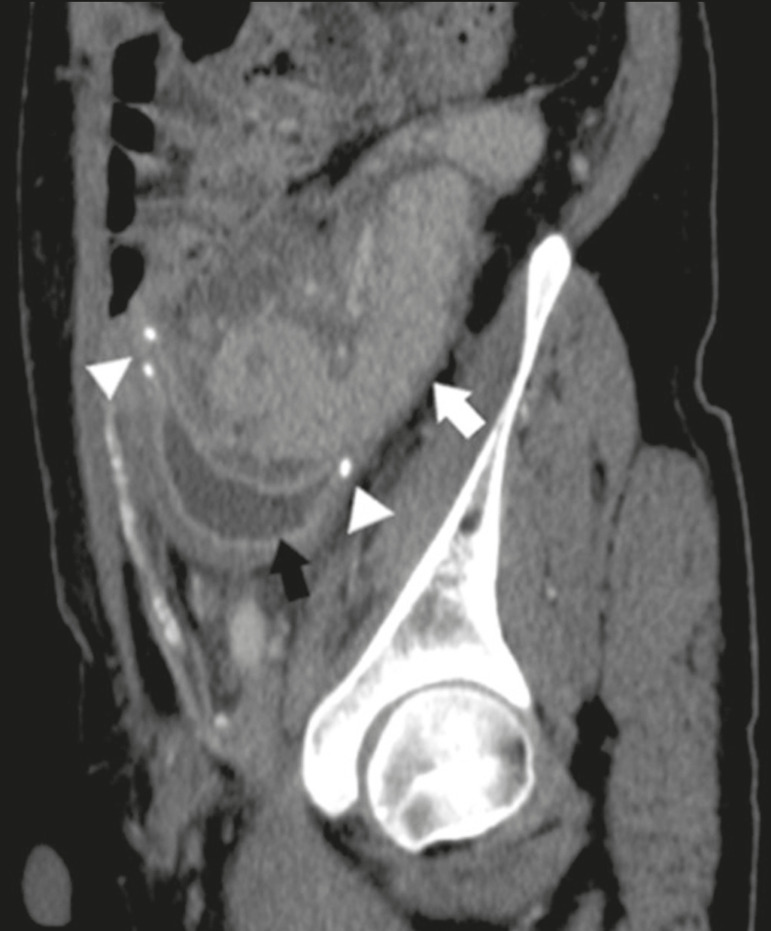
Sagittal reconstruction of a contrast-enhanced CT scan of a pancreatic graft with enteric drainage in the right iliac fossa (white arrow), showing the surgical staples (arrowheads) along the duodenal stump (black arrow), which is partially distended and may simulate a fluid collection. Surgical staples facilitate this differentiation, as well as helping locate the pancreatic graft.

The donor duodenum is usually collapsed and has thickened walls, which can be misinterpreted as a fluid collection when distended^(2)^. The surgical staples on either side of the duodenal stump may be useful for localization and differentiation (Figure 7).

**Figure 8:**
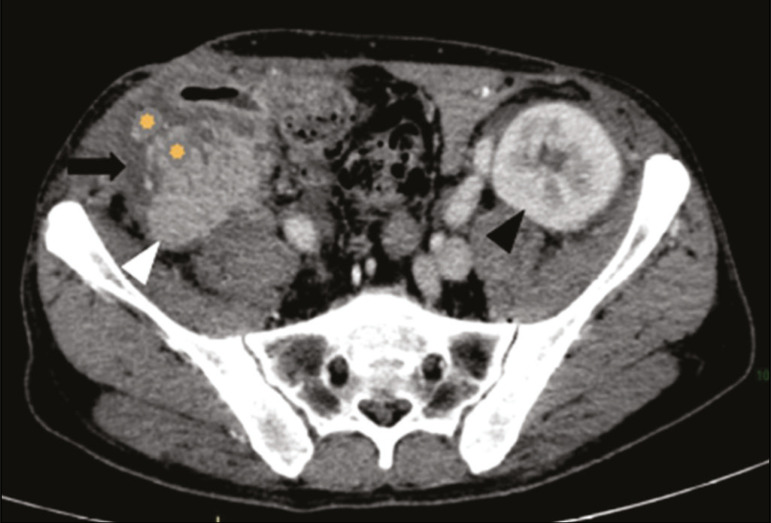
Contrast-enhanced axial CT scan, in the portal phase, of a patient in the postoperative period after simultaneous transplantation of a pancreas (white arrowhead) and a kidney (black arrowhead), presenting a small amount of peripancreatic fluid (arrow), in addition to adjacent lymph nodes (asterisks), usually without clinical significance, which may occasionally mimic some abnormality.

Other early postoperative findings, such as a limited amount of peripancreatic fluid (Figure 8), ectasia of the main pancreatic duct, and mild blurring of the peripancreatic fat, are usually self-limited and do not have clinical repercussions^(7)^.

## IMAGES OF COMPLICATIONS AFTER TRANSPLANTATIONS

As has previously been demonstrated^(1,2,7)^, early and late postoperative complications include vascular complications such as arterial and venous thrombosis; arterial stenosis; arteriovenous fistulae and pseudoaneurysms; intra-abdominal fluid collections; intestinal complications such as obstruction, dehiscence, and colitis; pancreatic graft complications, including pancreatitis and rejection; and lymphoproliferative disease after transplantation.

## VASCULAR COMPLICATIONS

**Figure 9:**
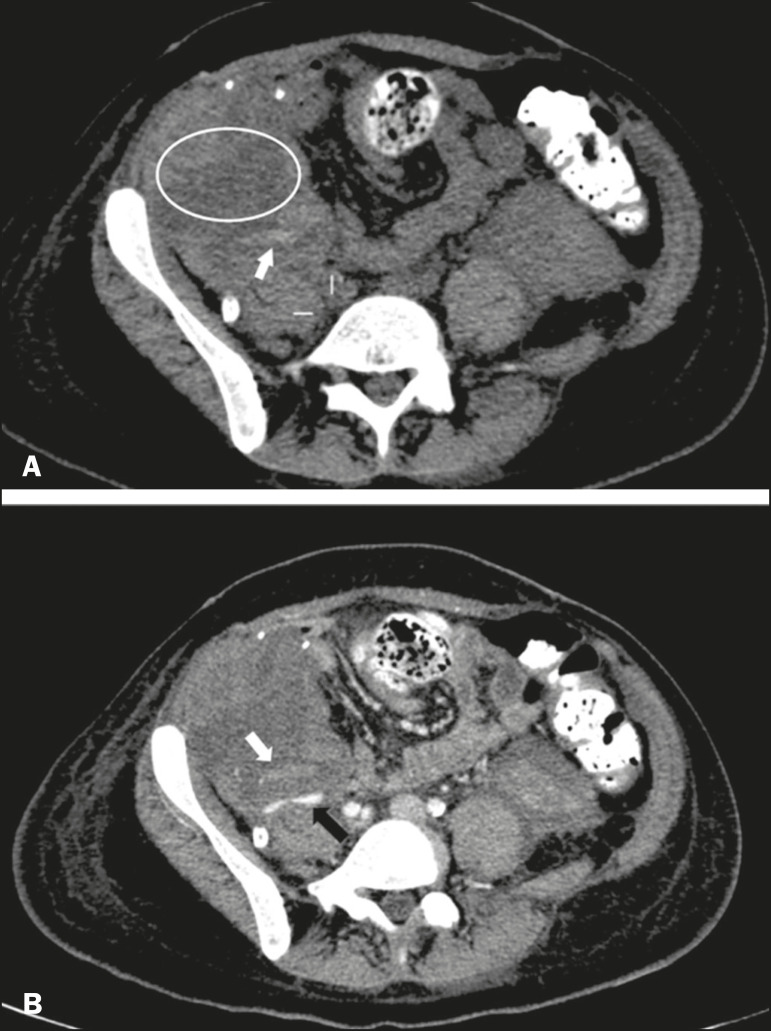
**A:** Unenhanced abdominal CT scan in patient who underwent SPKT, showing a linear image of a spontaneously hyperattenuating mass (arrow) suggestive of thrombosis. In addition, the pancreatic graft is heterogeneous, with hypoattenuating areas (ellipse). **B:** Contrast-enhanced abdominal CT scan, in the venous phase, of the same patient, showing arterial opacification (black arrow) and the absence of venous opacification (white arrow), confirming the venous thrombosis seen in the previous phases.

**Figure 10:**
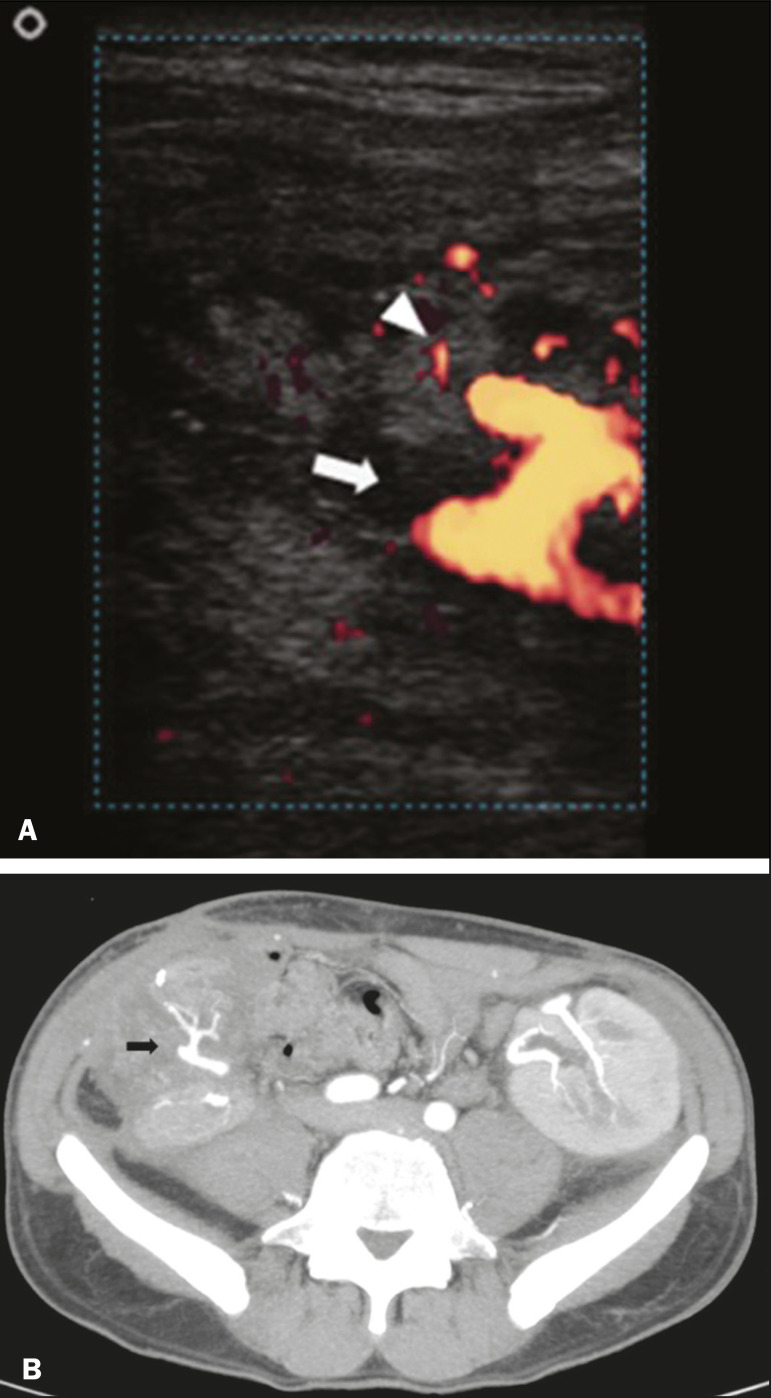
**A:** Cross-sectional image obtained by ultrasound in power Doppler mode, showing abrupt interruption of flow in the distal segment of the superior mesenteric artery (arrow) of the pancreatic graft, with adjacent collateral circulation (arrowhead), a finding consistent with arterial thrombosis. **B:** Correlation with contrast-enhanced CT in the same patient, with maximum intensity projection in the arterial phase, identifying the same abrupt interruption and a filling defect in the distal segment of the superior mesenteric artery of the pancreatic graft (arrow), confirming the arterial thrombosis suggested by the ultrasound finding.

Acute graft thrombosis is the leading cause of early transplant failure, accounting for 2-10% of cases, being more common than venous thrombosis^(1,2,7)^. An increase in graft volume with a reduction in attenuation or parenchymal heterogeneity can be observed (Figures 9A and 9B). The Doppler examination may reveal no flow in the vessel (Figure 10A) and, occasionally, in the entire parenchyma^(2,7)^. Poorly formed endoluminal thrombus (Figure 10B), with or without a decrease in or absence of parenchymal enhancement^(7)^, can be seen on contrast-enhanced CT or MRI.

Stenoses occur in any anastomotic site, are uncommon, and are characterized by high velocity/turbulence on ultrasound, usually confirmed with CT angiography or magnetic resonance angiography^(1,2)^. Pseudoaneurysms are associated with surgical trauma, biopsies, severe pancreatitis, or infection^(7)^. Rounded anechoic structures, adjacent to the vessels, are observed on ultrasound, the so-called “yin-yang” sign (a bidirectional, turbulent, swirling blood-flow pattern) being seen on Doppler ultrasound. Contrast-enhanced CT or MRI can identify saccular dilatation with enhancement similar to the adjacent vessel^(2,7)^. Arteriovenous fistula is another uncommon complication, usually being iatrogenic (after surgery or biopsy). Doppler ultrasound reveals a high speed/turbulent flow of low resistance in an arterial lumen that communicates with a vein, which presents a pulsatile flow^(2)^.

## INTRA-ABDOMINAL FLUID COLLECTIONS

**Figure 11:**
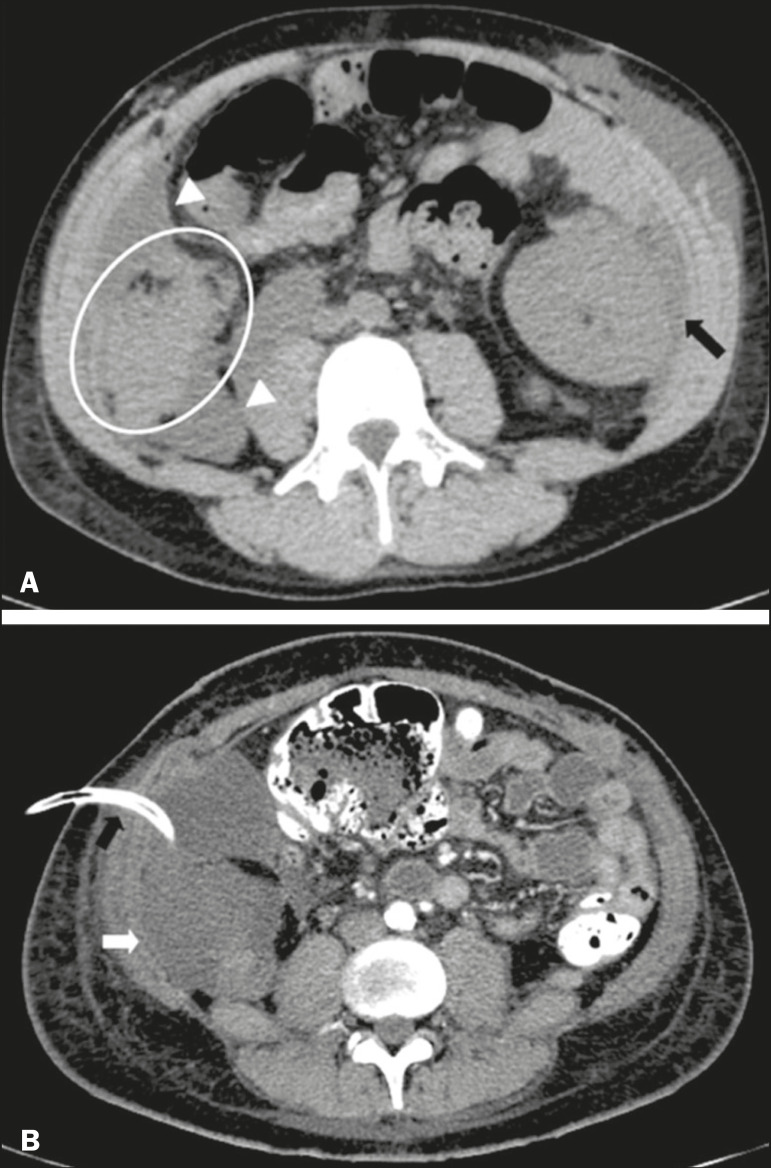
**A:** Unenhanced axial CT scan of the abdomen, showing fluid collections (arrowheads) adjacent to the pancreatic graft (ellipse). Note also the minimal amount of liquid adjacent to the renal graft on the left (black arrow). **B:** Fluid collection (white arrow) in the right iliac fossa of patient who underwent SPKT with percutaneous drainage (black arrow) seen on an axial CT scan of the abdomen with oral contrast.

Intra-abdominal fluid collections represent the most common complications associated with pancreas transplantation^(7)^. Most complications appear in the first month after the procedure and may manifest as seromas, hematomas, urinomas, abscesses, pseudocysts, or lymphoceles (Figure 11A). The nature of the fluid cannot usually be determined by imaging alone, and percutaneous drainage, as shown in Figure 11B, may therefore be essential for management of the case^(2,7)^.

## INTESTINAL COMPLICATIONS

**Figure 12:**
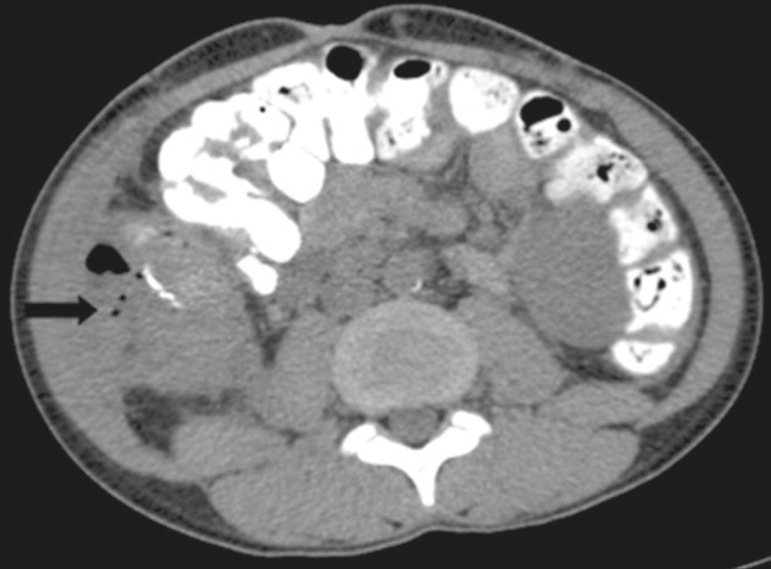
Axial CT with oral contrast of a patient in the early postoperative period of SPKT, with dehiscence of the anastomosis between the duodenal stump of the donor and the small intestine of the recipient, showing a fluid collection with gaseous foci adjacent to the topography anastomosis (arrow).

The most common intestinal complications are dehiscence and obstruction^(1,7)^, as illustrated in Figure 12. Obstruction usually occurs as a result of adherence or internal hernia, and CT is important to define the location of the obstruction^(1,2,7)^. Dehiscence commonly occurs in the intestinal anastomosis and may result in extravasation of pancreatic enzymes, predisposing to intra-abdominal infection^(1,2)^. Enterocutaneous fistulae may arise if dehiscence is not diagnosed and treated^(2)^. Therefore, the administration of oral contrast may be useful, because it can reveal direct or indirect signs of extravasation^(2,7)^.

## PANCREATIC GRAFT COMPLICATIONS

It is estimated that postoperative pancreatitis occurs in approximately 35% of pancreas transplant recipients^(2,7)^. Ultrasound can demonstrate an increase in graft volume, textural heterogeneity, or complications, such as fluid collections, pseudocysts, and necrosis^(1,2,7)^. Contrast-enhanced CT and MRI are fundamental for identifying the absence of regional or diffuse parenchymal enhancement in cases of necrotizing pancreatitis, as well as possible complications^(1,2,7)^.

Rejection (acute, subacute, or chronic) is the main cause of graft loss^(2,7)^. Although some imaging findings are suggestive of rejection, including changes in the size of the gland, signal intensity, and enhancement pattern, they are nonspecific and may also occur in pancreatitis and ischemia^(2,7)^. Therefore, the histopathological study continues to be the gold standard^(7)^.

## LYMPHOPROLIFERATIVE DISEASE AFTER TRANSPLANTATION

**Figure 13:**
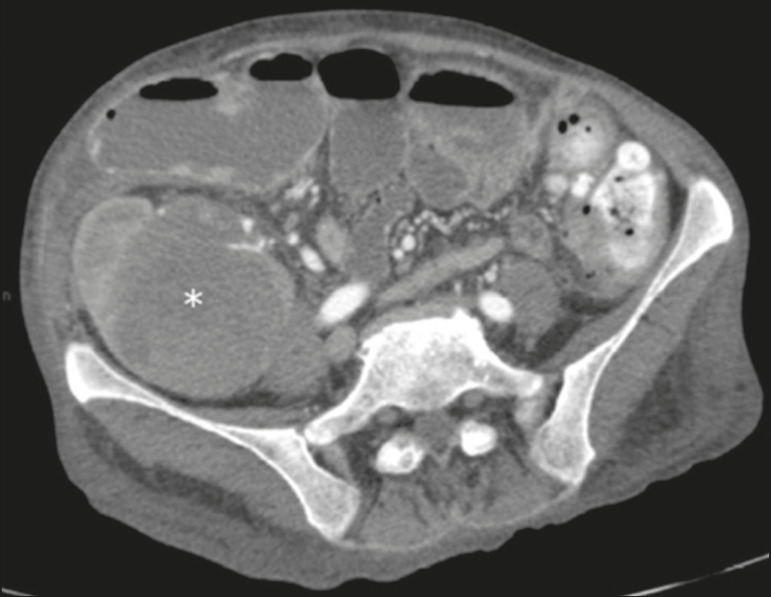
Contrast-enhanced axial CT of the abdomen, in the arterial phase, showing, at the level of the renal graft, a solid, slightly heterogeneous mass that is hypodense in relation to the renal parenchyma (asterisk), which was indicative of lymphoproliferative disease, after transplantation.

Lymphoproliferative disease, ranging from benign lymphoid hyperplasia to aggressive, malignant, and predominantly B-cell lymphoma, is a rare late complication of transplantation. The imaging spectrum includes focal masses and an increase in the volume of the graft, associated with lymphadenopathy^(2)^, as depicted in Figure 13.

## CONCLUSION

In advanced cases of diabetes, SPKT represents a viable therapeutic option. The most commonly used surgical technique involves the performance of vascular and enteric anastomoses. Radiologists should be aware of the characteristics of those anastomoses, as well as of their imaging aspects, which are fundamental for guiding the postoperative management of transplant recipients.

## References

[1] Tolat PP, Foley WD, Johnson C (2015). Pancreas transplant imaging: how I do it. Radiology.

[2] Vandermeer FQ, Manning MA, Frazier AA (2012). Imaging of whole-organ pancreas transplants. Radiographics.

[3] Redfield RR, Scalea JR, Odorico JS (2015). Simultaneous pancreas and kidney transplantation: current trends and future directions. Curr Opin Organ Transplant.

[4] Meirelles Júnior RF, Salvalaggio P, Pacheco-Silva A (2015). Pancreas transplantation: review. Einstein (Sao Paulo).

[5] Associação Brasileira de Transplante de Órgãos (2019). Dados numéricos da doação de órgãos e transplantes realizados por estado e instituição no período: janeiro/setembro - 2019.

[6] Papachristos S, Tavakoli A, Dhanda R (2019). Comparison of ipsilateral and contralateral simultaneous pancreas and kidney transplantation: a single-center analysis with 5-year outcome. Ann Transplant.

[7] Antunes N, Santos R, Almeida FG (2017). Pancreatic transplantation: what the radiologist needs to know. Acta Radiol Port.

[8] Sugi MD, Joshi G, Maddu KK (2019). Imaging of renal transplant complications throughout the life of the allograft: comprehensive multimodality review. Radiographics.

